# Relationships of Nutritional Factors and Agrochemical Exposure with Parkinson’s Disease in the Province of Brescia, Italy

**DOI:** 10.3390/ijerph19063309

**Published:** 2022-03-11

**Authors:** Michael Belingheri, Yueh-Hsiu Mathilda Chiu, Stefano Renzetti, Deepika Bhasin, Chi Wen, Donatella Placidi, Manuela Oppini, Loredana Covolo, Alessandro Padovani, Roberto G. Lucchini

**Affiliations:** 1 School of Medicine and Surgery, University of Milano-Bicocca, 20090 Monza, Italy; 2 Department of Environmental Medicine and Public Health, Icahn School of Medicine at Mount Sinai, New York, NY 10029, USA; mathilda.chiu@mssm.edu (Y.-H.M.C.); chi.wen@mssm.edu (C.W.); 3 Department of Medical and Surgical Specialties, Radiological Sciences and Public Health, University of Brescia, 25123 Brescia, Italy; stefano.renzetti@unibs.it (S.R.); donatella.placidi@unibs.it (D.P.); manuela.oppini@gmail.com (M.O.); loredana.covolo@unibs.it (L.C.); rlucchin@fiu.edu (R.G.L.); 4 Department of Surgery, Icahn School of Medicine at Mount Sinai, New York, NY 10029, USA; deepika.bhasin@mountsinai.org; 5 Department of Clinical and Experimental Sciences, University of Brescia, 25123 Brescia, Italy; alessandro.padovani@unibs.it; 6 Department of Environmental Health Sciences, School of Public Health and Social Work, Florida International University, Miami, FL 11200, USA

**Keywords:** Parkinson’s Disease, agricultural chemical exposure, nutritional factors, metals exposure, SNCA polymorphism

## Abstract

Environmental exposures to agrochemicals and nutritional factors may be associated with Parkinson’s Disease (PD). None of the studies to date has examined the combined effects of diet and agricultural chemical exposure together. To address these research gaps, we aimed to assess the association of nutritional factors and agrochemical exposure with the risk of PD. A hospital-based case-control study was conducted. Multivariable logistic regressions were used to estimate the association of nutritional and agrochemical exposures with PD, adjusting for gender, age, socio-economic status, head injury, family history, smoking, metals exposure, and α-synuclein gene polymorphism. Weighted Quantile Sum (WQS) regression was applied to examine the effect of dietary components as a mixture. We recruited 347 cases and 389 controls. Parent history of PD (OR = 4.15, 95%CI: 2.10, 8.20), metals exposure (OR = 2.50, 95%CI: 1.61–3.89), SNCA rs356219 polymorphism (OR = 1.39, 95%CI: 1.04–1.87 for TC vs. TT; OR = 2.17, 95%CI: 1.43–3.28 for CC vs. TT), agrochemical exposures (OR = 2.11, 95%CI: 1.41–3.16), and being born in the Brescia province (OR = 1.83, 95%CI: 1.17–2.90) were significantly associated with PD. Conversely, fish intake and coffee consumption had a protective effect. The study confirmed the role of environmental exposures in the genesis of PD. Fish intake and coffee consumption are protective factors even when agricultural chemical exposures exist. Genetic factors and metals exposure were confirmed as risk factors for PD.

## 1. Introduction 

Parkinson’s Disease (PD) is the second-most common neurodegenerative disorder after Alzheimer’s disease. The prevalence of PD is increasing due to an increase in life expectancy. The pathogenesis of PD is complex and multifactorial because it involves both genetic and non-genetic environmental factors, and the clinical heterogeneity of PD is probably due to the effect of different interactions between genes and environment or genes and genes [1,2,3,4,5]. 

In the last decades, several studies on genetic susceptibility factors have linked some single nucleotide polymorphisms (SNPs) to an increased risk of PD [1,4,5,6,7]. Among them, the α-synuclein (SNCA) gene polymorphisms have been identified as associated with PD risk [4,6].

Aside from the genetic background, environmental factors—including occupational exposures—have gained importance in the etiology of PD [8,9]. Particularly, environmental exposures to heavy metals and agrochemicals may be associated with PD risk [10,11,12,13,14,15]. Several studies have described the role of heavy metals in neurodegenerative diseases and the relationship between chronic exposure to metals and the increased risk of PD [16]. Similarly, exposure to agricultural chemicals is known for its neurotoxic impact and the consequent neurodegenerative damage, especially targeting the dopamine system [13]. Several studies have reported the association between chronic exposure to organophosphates, pesticides, fertilizers, and herbicides and the risk of PD [10,13,14,15].

Nutrition also plays an important role in the pathogenesis of PD. Some studies suggested that nutritional factors may have both neuroprotective and neurodegenerative effects resulting in the onset of PD [17,18,19]. Dairy product consumption, red meat, and animal fat may increase the risk of PD, while high fish intake, fruit, vegetables, and caffeine consumption are suggested to be inversely associated with PD [20,21,22,23,24]. Logroscino et al. conducted a population-based case-control study among 110 cases and 287 controls in the USA, and they found an increased risk of PD with increased dietary animal fat [25]. Anderson et al. also found that dietary intake of animal fat increased the risk of PD, while no increased risk was found for fruit and vegetables [25]. In another case-control study in southeastern Sweden among 113 cases and 263 controls, Fall et al. found a reduced risk of PD with increased niacin-rich food, whereas an increased risk of PD was observed when exposed to pesticides [26]. 

Further, several studies have demonstrated the independent effects of nutritional and agricultural chemical exposures in the occurrence of PD [9,24,25,26,27,28,29,30]. However, most studies have relatively small sample sizes, and, to our knowledge, none of the studies to date has examined the combined effects of both nutritional and agricultural chemical factors.

To address these research gaps, we conducted a hospital-based case-control study in the province of Brescia, Northern Italy. We aimed to assess the association of nutritional factors and agrochemical exposure with the risk of PD. We also included data on metals exposure and genotyping analysis for a specific SNP of SNCA (rs356219), known to be associated with PD risk [7]. The present study is a part of a larger study about PD and parkinsonism, conducted in a highly polluted and industrialized area of Italy [7,11,31,32] with documented variations in environmental exposures as well as genetic variations [7,11,33,34,35,36,37].

## 2. Materials and Methods

### 2.1. Study Population

The study population consisted of 876 individuals (age range 40–94 years) accessing four local hospitals located in the Brescia province—ASST-Spedali Civili of Brescia, Esine Hospital of Valcamonica, Poliambulanza Foundation, and Ancelle Domus Salutis. These centers were specialized in movement disorders. Data were collected in 2012–2015.

The case definition included not only the idiopathic PD but a broader classification of parkinsonism. Cases were defined by the presence of at least two of the following features: bradykinesia, akinesia, rigidity, tremor, postural instability [38]. Patients with a previous diagnosis of dementia with Lewy bodies [39], progressive supranuclear palsy [40], corticobasal syndrome [41], and multiple system atrophy [42] were included. Patients with a previous diagnosis of iatrogenic parkinsonism or traumatic parkinsonism, as well as parkinsonism related to brain tumors or encephalitis were excluded. Controls were selected randomly from other departments (including dermatology, internal medicine, neurosurgery, ophthalmology, and orthopedics) in the same hospitals to provide a similar geographical distribution to the cases. The controls were matched to cases based on race, sex, and age (±5 year), and those with a previous diagnosis of neurological or psychiatric disease were excluded. All subjects underwent blood sampling for DNA analysis upon enrollment.

The study protocol was reviewed and approved by the Ethical Committee of the Civil Hospital of Brescia, Italy (ref. NP 1189 PARKGENEAMB). The patients/participants received written and oral explanations about the research and provided their written informed consent to participate in the study.

A questionnaire was administered to the participants. The information gathered from the questionnaire included demographics, province of birth and occupational history, occupational and environmental exposures, dietary intake, symptoms and diagnosis, medical history, history of medications, family history of PD, and lifestyle history, such as smoking and drinking status. All information gathered with the questionnaire was focused on the timeline before the diagnosis of PD.

For the analysis, we included 736 individuals (347 cases and 389 controls) that had complete data on dietary intake and parental history of PD. The excluded individuals included those missing parental history of PD (10.7%) as well as the other missing values comprised of 2.3% for vegetable intake, 1.8% for fruit intake, 0.1% for carbs intake, 1.6% for chemical exposures, 0.9% for fruit intake, and 0.3% for head injury.

### 2.2. Nutritional Exposure

To assess nutritional exposure, we utilized a food frequency questionnaire specifically developed for this study based on the eating habits of the Italian population [43]. The questionnaire was weighted on portion size and focused specifically on dietary consumption of fish, vegetables, fruit, dairy products, carbs, coffee, and red or white meat intake. We combined the various dietary consumptions into broad categories: (i) large fish, seafood, and local fish included as fish intake; (ii) vegetables in any form, including cooked and uncooked vegetables; (iii) fruit consumption included fruit in juice form or as a whole fruit; (iv) dairy products included both fresh and seasoned cheese, as well as milk and yogurt; (v) bread, pasta, and bread sticks were combined to indicate carbohydrates (carbs) intake. The intake of red meat and white meat were considered separately. The food consumption in the questionnaire was referred to serving size, and the frequency was reported as: ‘Never or less than 1 time a month; 1–3 times a month; once a week; 2–4 times a week; 5–6 times a week; once a day; 2–3 times a day; 4 or more times a day.” The frequency of food consumption was then converted to a continuous score (servings per month; see Supplemental Materials), which was used in the analysis.

### 2.3. Exposures to Agricultural Chemical and Metals

Participants reported their potential workplace exposure to agrochemicals listed in the questionnaire, including Herbicide, Glyphosate, Paraquat, Carbaryll, Chlorpyrifos, DDT, Heptachlor, Kepone, Malathion, Parathion, and Rotenone. Chemicals for potential home or non-occupational exposure included Insecticide, DDT, Herbicide, and Fungicide, either prepared by the individual or as available from commercial products. Because of low frequencies of the exposure to a single chemical, the agricultural chemical exposure was combined into a dichotomous variable, “yes, having exposed to agrochemicals prior to diagnosis” versus “never exposed to”.

Exposure to metals was considered as a dichotomous variable, considering an overall exposure measure, by identifying participants with at least one occupational exposure to metals in their job life. The questionnaire included aluminum, antimony, arsenic, beryllium, cadmium, copper, chromium, gallium, magnesium, manganese, mercury, nickel, and lead.

### 2.4. Genotyping

Both cases and controls were tested for genotyping, a DNA analysis for the assessment of specific polymorphic variants. DNA was extracted from 0.2 mL of peripheral whole blood samples using the QIAamp DNA Blood Mini kit (Qiagen, Hilden, Germany). Genotyping was performed for synuclein alpha (SNCA: rs356219) with TaqMan real-time PCR. The variant rs356129 was considered since it is known to be associated with the risk of PD, as confirmed by the results of the main study to which this one is part [7]. The effect of the genetic polymorphism was modeled assuming a co-dominant model. The homozygous genotype TT was considered as the reference level (lowest risk class for PD), the heterozygous genotype TC as intermediate risk class, and the homozygous genotype CC as the highest risk class for PD [7].

For quality control of genotyping data, >5% of samples were re-analyzed in a separate round of experiments with a 100% agreement between duplicates [7].

### 2.5. Covariates

Family history of PD was determined based on paternal and maternal PD status. Socio-economic status (SES) was defined using lifelong occupation history described by the International Standard Classification of Occupations (ISCO) and the International Standard Industrial Classification (ISIC) codes. Since we did not have information about the income of participants, we estimated the SES considering the occupation history as an approximate indicator of the economic and social position. We used the earliest occupational history based on ISCO code before the PD diagnosis. If the ISCO code was missing, then ISIC code at the same time period was used. If both codes were missing, the next occupational history was used. The broad categories of occupations were categorized as high, medium, and low SES (see Supplemental Materials, Table S1). Information about the birth location was also collected and categorized based on the province: within the province of Brescia or outside. This categorization allowed us to consider environmental exposure since Brescia has been one of the most polluted industrial areas in Italy since the last century [11].

### 2.6. Statistical Analysis

For the descriptive analysis, we used the *t*-test to compare between PD cases and controls on the continuous variables and the Chi-square test for the categorical variables, such as gender, SES, parental history of PD, chemical exposures, coffee intake. We then conducted univariate and multivariable logistic regression models to examine the association between these potential predictors and PD. All multivariable models were adjusted by factors that have been associated with PD risk, including family history of PD, history of head injury, smoking, as well as demographics, such as age, sex, socio-economic status. To account for the potential geographical impact of birth province on fruit and vegetable due to potential industrial contaminations in different agricultural areas, we further conducted an interaction model by additionally including interaction terms between these two variables and birth province, for which we standardized fruit and vegetable intakes for a better interpretation of the results. Finally, we conducted Weighted Quantile Sum (WQS) regressions [44,45] to examine the effect of the diet/nutrient “mixture”. WQS regression is a recent statistical method that allows the estimation of the overall effect of the mixture on the considered health outcome through the estimate of a weighted index. The empirical weights estimated by the WQS model represent the contribution of each element included in the mixture to the association with the outcome. In our study, the mixture exposure is identified by the nutrients included in the diet. Further, it has been shown that WQS regression performs well with highly dimensional and correlated data. The continuous score of dietary intake frequency was ranked into quartiles. Two separate WQS models were conducted to examine the associations when constraining the effect of diet mixture on PD as either in a positive or negative direction. We applied WQS regressions for binary outcomes with repeated holdout validation [44,45]. Briefly, the WQS model is a novel statistical method developed in the context of exposure to environmental mixtures that allows the measure of the effect of a set of elements (chemicals, nutrients, etc.) on the outcome of interest. Through the repeated holdout method, we were able to overcome the problem of unstable estimates and unrepresentative partitions caused by the split in training and validation sets that occurs in the WQS procedure. In this case, we created 100 random training-validation partitions, and for each partition, we applied 100 bootstraps to estimate the WQS weights. The food consumption frequency was split into quartiles to have the same scale for the different elements in the mixture. For all models, the presence of influential values and multicollinearity was checked through the Cook’s distance and the Variance Inflation factor (VIF), respectively. Finally, as there is a small portion (12.5%) of study subjects born outside of the Brescia province, we also performed a sensitivity analysis, only restricting to subjects born in the Brescia province. The data analysis was performed using SAS (version 9.4, SAS Institute Inc., Cary, NC, USA) and R (version 4.0.2); the R package gWQS (version 3.0.3) was used to apply the repeated holdout WQS to the data.

## 3. Results

There were 347 participants with PD (cases) and 389 without PD (controls). The main characteristics of cases and controls are presented in Table 1. The cases were slightly older than controls (mean of 71.9 vs. 69.5 years, respectively, *p* < 0.001). Parental history for PD, agricultural chemical exposure, metals exposure, and being born in the province of Brescia were, in general, more prevalent among cases than controls.

The results from the multivariable logistic regression models examining the relationships of environmental and nutritional factors with PD are shown in Table 2.

All models showed no influential values, and all covariates had a VIF below 2.4, meaning that there was no multicollinearity. Model 1 shows the effect estimates without including interaction with the province of birth. We found that the parental history of PD (OR = 3.58; 95%CI: 1.58, 8.94), exposure to metals (OR = 2.34; 95%CI: 1.31, 4.27), polymorphism rs356219 in the SNCA gene (OR = 2.10, 95%CI: 1.30, 3.43 for CC vs. TT), exposure to agricultural chemicals (OR = 1.98; 95%CI: 1.28, 3.10), and older age (OR = 1.03; 95%CI: 1.01, 1.05, per year increase) were associated with an increased risk of developing PD. A positive association also resulted with the consumption of fruit (OR = 1.27; 95%CI: 1.02, 1.59) and vegetables (OR = 1.01; 95%CI: 1.00, 1.02). Being born in the province of Brescia was associated with an increased risk of PD (OR = 1.73; 95%CI: 1.05, 2.90). A marginally significant protective effect was seen for fish consumption (OR = 0.98; 95%CI: 0.96, 1.00). To further investigate the potential geographical effects of agricultural chemical contaminations on fruit and vegetables, we conducted Model 2, which additionally included the interaction terms between the province of birth and the consumption of fruit and vegetables. The positive relationships of vegetable and fruit intakes with PD observed in Model 1 were no longer observed after including the interaction between these variables and the province of birth in Model 2. The associations were similar to Model 1 in terms of the positive associations with parental history of PD, exposure to metals, polymorphism rs356219 in the SNCA gene, exposure to agricultural chemicals, and older age, as well as the protective effect of fish consumption. We did not find any association between the risk of PD and other nutritional exposures, such as carbs, dairy consumption, and red or white meat intake. Furthermore, we did not find any association with history of head injury, smoking, or socio-economic status.

The results of WQS regressions estimating the association between diet mixture and PD are shown in Table 3. We ran two separate WQS models, constraining the effect of diet mixture on PD as either in a positive direction (Table 3, Model *P*) or negative direction (Table 3, Model *N*).

**Figure 1:**
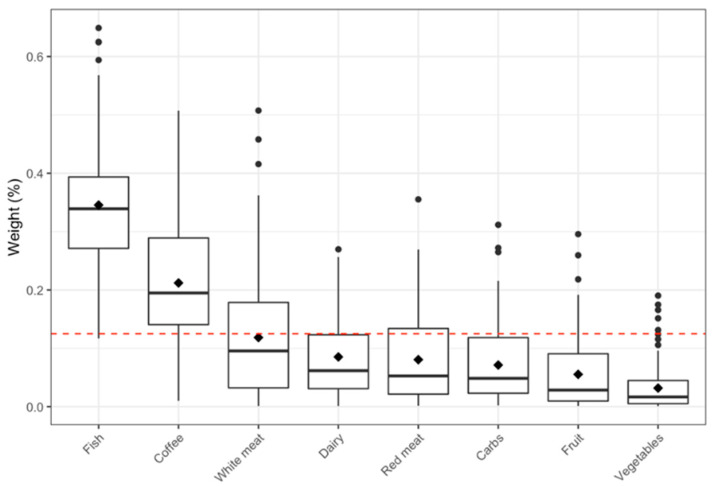
Boxplots of the weights estimated through the Weighted Quantile Sum (WQS) regression representing the distribution generated by the 100 repeated holdout validations for each element considered in the mixture. The set of weights are those associated with the nutrient mixture index with a protective effect on PD. The diamond corresponds to the weight mean value for each nutrient. The red dashed line is the prespecified threshold (equal to the inverse of the number of elements in the mixture: 1/8) to establish the weights different from 0.

A statistically significant effect estimate of the diet mixture on the PD was observed only for the model constraining the diet mixture as negative (protective effect; OR of total diet WQS index = 0.72, 95%CI: 0.53–0.99). When looking at the WQS weights associated with each nutrient (Figure 1), the most important protective elements (those with higher weights) were fish intake (34.5%) and coffee consumption (21.2%) (Supplemental Materials, Table S2), indicating that an increase in fish or coffee intake may be the most important contributors on the protective association between dietary mixture and PD. These were the only two significant components if we consider the inverse of the number of nutrients in the mixture (in our case 1/8 = 0.125) as a cutoff that corresponds to the values of a non-informative scenario. Parental history of PD, exposure to metals, polymorphism rs356219 in the SNCA gene, exposure to agricultural chemicals, and age remained significantly associated with PD in the WQS analysis.

For the sensitivity analysis restricted to subjects born in the Brescia province, the results were materially unchanged in terms of significance in *t*-test, chi-squared tests, and logistic regression. We only observed a difference in WQS regression where the protective effect of the nutrient mixture did not reach statistical significance after restricting to subjects born in Brescia province, but this was mainly due to a larger confidence interval (i.e., higher standard error likely due to reduced sample size) as the OR estimate for WQS index of the nutrition mixture remained similar to that from the full sample model (negative WQS index OR = 0.79 (95%CI: 0.53–1.19) when restricted to those born in Brescia, as compared to OR = 0.72 (95%CI: 0.53–0.99) in the full sample).

## 4. Discussion

The findings of this study confirmed the role of some genetic and environmental factors in the pathogenesis of PD, as previously described in other studies on these topics [5,9,10,11,12,13,14,15,16,17,22,23,24]. Our results indicated that agricultural chemical exposures, metals exposure, SNCA rs356219 polymorphism, parental history of PD, age, and being born in the Brescia province were significantly positively associated with increased PD, whereas coffee consumption and fish intake resulted as protective nutritional factors for PD.

The aim of the study was to assess the association between nutritional exposures and agricultural chemical exposures and the risk of PD. One of the major concerns of the previous studies investigating these relationships was that there is limited literature on this topic with adequate sample size and proper adjustment for potential confounders [10,26]. Therefore, we included other factors that are well-known to be associated with the risk of PD, as potential confounders, such as SNCA polymorphism and metals exposure [5,7].

Previous studies suggested that vegetables and fruit may have a neuroprotective effect on PD [22,23,24]. Interestingly, however, in Model 1 of our multivariable logistic regression (Table 2), the results showed that fish intake was protective for PD, while vegetables and fruit consumption was associated with an increased risk of PD. One of the possible explanations of this unexpected result in Model 1 may be due to the consumption of unwashed fruit and vegetables, resulting in the oral absorption of agrochemicals. The soil of the Brescia province in is known to be highly contaminated by manganese and other metals as a product of century-long ferroalloy activities [11,12,33,34,46,47,48]. Metals such as manganese have been linked to neurodegenerative effects increasing the risk of PD. The environmental pollution may also explain the association between being born in Brescia province and the increased risk of PD. This has also been implicated in the results of our interaction model (Model 2 in Table 2), in which we observed that both fruit and vegetables were no longer the significant positive predictors of PD after additionally including the interaction terms between the province of birth (Brescia vs. other locations) and vegetables and fruit consumption into the model, suggesting the possibility of agriculturally contaminated food as the reason driving the positive association we saw in Model 1. Notably, Sääksjärvi et al. also reported a similar observation where they saw an association between increased consumption of fruit and elevated risk of PD. They assumed that pesticides in farming may increase the risk of PD, reducing or masking the protective role of vegetables and fruit consumption [49].

The role of diet in the pathogenesis of PD is interesting because nutrients may have neurodegenerative effects, but other nutrients may have neuroprotective effects, thus reducing the risk of PD. When we considered a mixture approach of the nutrients through the application of the WQS regression, we found a protective effect of fish and coffee intake. These important findings are consistent with previous observations from other studies, which reported a negative association between the consumption of fish and coffee and the risk of PD [20,21,23,24]. Fish, in general, is rich in unsaturated fatty acids, which have protective properties and are associated with a low risk of PD [50]. Caffeine is known as a nervous system stimulant able to interact with adenosine receptors, improving motor deficits [21]. Additionally, compared to previous studies, the application of WQS to these data allowed the estimation of the weighted total effect of a diet mixture on PD. This is additional information that this method allows data to be extrapolated from considering nutrients as a whole and not just estimating the single effect that each element has on the outcome.

However, although fish consumption is known to have a protective effect for neurodegenerative disease, it is important to consider that information on fish consumption is dated back a few years (before the diagnosis); therefore, it may relate to a time when fish contamination was lower than today. The increasing water pollution may contaminate fish, reducing or masking the protective role of fish consumption, similarly to what happens for the contamination of vegetables and fruit.

Other nutrients have been studied to assess their role in the pathogenesis of PD. Some of the previous studies have linked an increased risk of PD with animal fat [24,25,27], as well as with dairy products consumption [20,51]. However, our analysis could not confirm this association. Further studies with more detailed meat and dairy intake information are needed to tease out the micronutrients in meat and dairy that are most associated with PD.

The exposure to agricultural chemicals—organochlorines, pesticides, fertilizers, herbicides—has been found to be associated with an increased risk of disease. This finding confirms the neurodegenerative effects of some agrochemicals, as already reported in previous studies in which their role in the pathogenesis of PD has been described [10,13,14,29]. Furthermore, our analysis included other factors that were known to be associated with the risk of PD, such as metals exposure and SNCA polymorphism (rs356219). The results related to these factors are consistent with previous studies. Exposure to heavy metals, such as manganese and lead, was confirmed as a risk factor for PD, and the presence of SNCA polymorphism was confirmed to be associated with an increased risk of disease [4,5,9].

The strengths of this study include the homogeneity of the study population, the availability of data on a large set of known risk factors for PD [7], and the application of WQS regression to better characterize the integrated effect of nutrients on PD. We also acknowledge potential limitations. There is some possibility of recall bias, as with any retrospective study using questionnaires [7]. The questions asked about events that occurred in the past, such as dietary intake, residential history, and past lifestyle information. Nonetheless, since the data collection was similar across cases and controls, the bias is likely to be non-differential. In addition, the measurement for metal exposure and agrochemicals was a crude yes or no based on a list of commonly used occupational and non-occupational chemicals, without a specific measurement of the exposure. Further biomarker analyses in this cohort with a more defined exposure timepoint will allow us to better assess the exposure reconstruction.

Finally, despite adjusting for all the main risk factors (including metals and SNCA polymorphism), a potential issue of residual confounding may remain. For example, while we adjusted for the province of birth (Brescia province as a higher polluted area vs. other regions), we were not able to distinguish among different subareas within the Brescia province. Finally, it is possible that our results might not be generalizable to populations with lower environmental pollution levels or different genetic origins.

In conclusion, the study confirmed the role of environmental exposures in the genesis of PD. Nutritional factors, agricultural chemicals and occupational metals exposure were confirmed as important determinants that may increase (or decrease) the risk of PD. Whilst agrochemicals and metals were associated with an increased risk of disease, the consumption of fish and coffee resulted as protective nutritional factors. Genetic factors (family history of PD, SNCA rs356219 polymorphism) were confirmed as risk factors for PD. Further prospective longitudinal studies are needed to better understand the interrelation between nutritional factors and toxic exposure in the origin of PD.

## Figures and Tables

**Table 1:** Characteristics distribution of the overall population and divided by participants with and without PD. *p*-value of *t*-test and chi-squared test were included to test the difference between No PD and PD groups for continuous and categorical variables.

	No PD (*N* = 389)	PD (*N* = 347)	Total (*N* = 736)	*p*-Value
**Age**				<0.001
Mean (SD)	69.5 (9.7)	71.9 (9.6)	70.6 (9.7)	
**Sex**				0.754
Female	158 (40.6%)	137 (39.5%)	295 (40.1%)	
Male	231 (59.4%)	210 (60.5%)	441 (59.9%)	
**SES**				0.792
Low	254 (65.3%)	232 (66.9%)	486 (66.0%)	
Medium	83 (21.3%)	67 (19.3%)	150 (20.4%)	
High	52 (13.4%)	48 (13.8%)	100 (13.6%)	
**Parent history of PD**				<0.001
No	381 (97.9%)	322 (92.8%)	703 (95.5%)	
Yes	8 (2.1%)	25 (7.2%)	33 (4.5%)	
**Agricultural Chemical exposure**				0.001
No	342 (87.9%)	275 (79.3%)	617 (83.8%)	
Yes	47 (12.1%)	72 (20.7%)	119 (16.2%)	
**Head injury**				0.490
No	340 (87.4%)	309 (89.0%)	649 (88.2%)	
Yes	49 (12.6%)	38 (11.0%)	87 (11.8%)	
**Ever smoke**				0.153
No	214 (55.0%)	209 (60.2%)	423 (57.5%)	
Yes	175 (45.0%)	138 (39.8%)	313 (42.5%)	
**Province of Birth**				0.011
Brescia	329 (84.6%)	315 (90.8%)	644 (87.5%)	
Other	60 (15.4%)	32 (9.2%)	92 (12.5%)	
**Metal exposure**				0.006
No	354 (93.7%)	301 (87.8%)	655 (90.8%)	
Yes	24 (6.3%)	42 (12.2%)	66 (9.2%)	
**SNCA rs356219**				0.004
TT	167 (43.9%)	113 (34.1%)	280 (39.4%)	
TC	167 (43.9%)	153 (46.2%)	320 (45.0%)	
CC	46 (12.1%)	65 (19.6%)	111 (15.6%)	
**Coffee intake**				0.067
Low	152 (39.1%)	164 (47.3%)	316 (42.9%)	
Medium	191 (49.1%)	143 (41.2%)	334 (45.4%)	
High	46 (11.8%)	40 (11.5%)	86 (11.7%)	
**Vegetable intake**				0.041
Low	114 (29.3%)	102 (29.4%)	216 (29.3%)	
Medium-Low	106 (27.2%)	82 (23.6%)	188 (25.5%)	
Medium-High	129 (33.2%)	103 (29.7%)	232 (31.5%)	
High	40 (10.3%)	60 (17.3%)	100 (13.6%)	
**Fruit intake**				0.139
Low	339 (87.1%)	289 (83.3%)	628 (85.3%)	
High	50 (12.9%)	58 (16.7%)	108 (14.7%)	
**Fish intake**				0.002
Low	80 (20.6%)	99 (28.5%)	179 (24.3%)	
Medium-Low	94 (24.2%)	102 (29.4%)	196 (26.6%)	
Medium-High	106 (27.2%)	81 (23.3%)	187 (25.4%)	
High	109 (28.0%)	65 (18.7%)	174 (23.6%)	
**Red meat intake**				0.336
Low	151 (38.8%)	120 (34.6%)	271 (36.8%)	
Medium	208 (53.5%)	192 (55.3%)	400 (54.3%)	
High	30 (7.7%)	35 (10.1%)	65 (8.8%)	
**White meat intake**				0.367
Low	349 (89.7%)	304 (87.6%)	653 (88.7%)	
High	40 (10.3%)	43 (12.4%)	83 (11.3%)	
**Carbs intake**				0.521
Low	121 (31.1%)	106 (30.5%)	227 (30.8%)	
Medium	201 (51.7%)	170 (49.0%)	371 (50.4%)	
High	67 (17.2%)	71 (20.5%)	138 (18.8%)	
**Dairy intake**				0.264
Low	114 (29.3%)	83 (23.9%)	197 (26.8%)	
Medium-Low	104 (26.7%)	91 (26.2%)	195 (26.5%)	
Medium-High	116 (29.8%)	124 (35.7%)	240 (32.6%)	
High	55 (14.1%)	49 (14.1%)	104 (14.1%)	

**Table 2:** Logistic regression results demonstrating the odds ratios (OR), 95% Confidence Intervals (CI), and *p*-values for the association between environmental, genetic, and dietary predictors and PD.

	Model 1			Model 2 ^a^		
Predictors	OR	95%CI	*p*-Value	OR	95%CI	*p*-Value
Age	1.03	1.01–1.05	<0.001	1.03	1.01–1.05	<0.001
Male	0.98	0.69–1.40	0.929	0.99	0.69–1.41	0.947
SES Medium vs. Low	1.12	0.74–1.71	0.586	1.12	0.74–1.71	0.596
SES High vs. Low	1.14	0.70–1.86	0.603	1.11	0.68–1.81	0.674
Parental PD history	3.58	1.58–8.94	0.004	3.64	1.59–9.16	0.003
Head Injury	0.88	0.52–1.46	0.611	0.88	0.53–1.47	0.63
Ever smoked	0.81	0.57–1.15	0.234	0.81	0.57–1.14	0.228
SNCA rs356219 (TC vs. TT)	1.32	0.93–1.87	0.125	1.32	0.93–1.87	0.124
SNCA rs356219 (CC vs. TT)	2.1	1.30–3.43	0.003	2.09	1.29–3.41	0.003
Agricultural chemical exposure	1.98	1.28–3.10	0.003	1.95	1.25–3.05	0.003
Metal exposure (Yes vs. No)	2.34	1.31–4.27	0.004	2.33	1.30–4.25	0.005
Coffee	1	0.99–1.00	0.109	1	0.99–1.00	0.105
Fish	0.98	0.96–1.00	0.06	0.98	0.96–1.00	0.066
Fruit	1.27	1.02–1.59	0.036	0.94	0.58–1.49	0.807
Vegetables	1.01	1.00–1.02	0.018	1.32	0.81–2.16	0.268
White meat	1.01	0.98–1.04	0.432	1.01	0.98–1.04	0.432
Red meat	1.01	0.99–1.04	0.315	1.01	0.99–1.04	0.291
Dairy	1	0.99–1.01	0.849	1	0.99–1.01	0.838
Carbs	1	1.00–1.01	0.586	1	1.00–1.01	0.579
Born in Brescia (BS)	1.73	1.05–2.90	0.035	1.69	1.02–2.84	0.043
(Fruit and BS) vs. Other				1.31	0.81–2.21	0.286
(Vegetables and BS) vs. Other				0.93	0.55–1.55	0.773

^a^ Model 2 additionally includes the interaction terms between fruit and vegetable consumptions with place of birth (Brescia vs. other).

**Table 3:** Weighted Quantile Sum (WQS) regression examining the relationships of diet mixture and environmental and genetic factors with PD. Odds ratios (OR) and 95% confidence intervals (CI) are shown.

	Model *P* ^a^		Model *N* ^a^	
	OR	95%CI	OR	95%CI
WQS index for diet mixture ^b^	1.305	(0.88, 1.936)	0.721	(0.525, 0.991)
Age	1.034	(1.019, 1.049)	1.031	(1.016, 1.046)
Males vs. Females	0.98	(0.764, 1.258)	0.979	(0.757, 1.265)
SES Medium vs. Low	1.135	(0.774, 1.663)	1.138	(0.776, 1.669)
SES High vs. Low	1.13	(0.766, 1.668)	1.182	(0.795, 1.758)
Parent PD history	4.165	(2.136, 8.12)	4.145	(2.096, 8.196)
Head Injury	0.883	(0.58, 1.344)	0.881	(0.578, 1.343)
Ever smoked	0.767	(0.565, 1.041)	0.759	(0.559, 1.031)
Agricultural chemical exposure	1.843	(1.173, 2.897)	2.113	(1.413, 3.159)
Metal exposure	2.501	(1.607, 3.893)	2.504	(1.610, 3.893)
Born in Brescia vs. others	1.843	(1.173, 2.897)	1.825	(1.169, 2.848)
SNCA rs356219				
TC vs. TT	1.396	(1.036, 1.88)	1.393	(1.037, 1.871)
CC vs. TT	2.138	(1.417, 3.225)	2.17	(1.434, 3.284)

^a^ This table demonstrates the results from the WQS regressions constraining the direction of the diet mixture on PD as either positive (Model *P*) or negative (Model *N*). The WQS index for diet mixture was only significant when the mixture effect direction was constraining to negative (Model *N*). The dietary components included in the WQS mixture are coffee, fish, white meat, red meat, dairy, carbs, fruit, and vegetables. ^b^ The estimated weights of each of the dietary components in the WQS index are shown in Figure 1.

## Supplementary Materials

The following supporting information can be downloaded at: https://www.mdpi.com/article/10.3390/ijerph19063309/s1, Table S1: Categories of socio-economic status (SES) based on the International Standard Classification of Occupations (ISCO) codes; Table S2: Average weight estimated for each nutrient over the 100 repeated holdout WQS estimates.
