# Supplementary material for: Relationships of Nutritional Factors and Agrochemical Exposure with Parkinson’s Disease in the Province of Brescia, Italy
Source: Int J Environ Res Public Health. 2022 Mar 11;19(6):3309. doi: 10.3390/ijerph19063309 (PMC8954923; doi:10.3390/ijerph19063309)
Supplement: Supplementary file 1 [file ijerph-19-03309-s001.zip › ijerph-1537881-supplementary.pdf]

SUPPLEMENTARY MATERIAL

The frequency of food consumption was converted into a continuous variable based on estimated frequency per month (each time = 1 serving). Below are the conversion equations:

1-3 times a month = 2 times a month

1 time a week = 4 times a month

2-4 times a week = 3*4 times a month = 12 times a month

5-6 times a week = 5*4 times a month = 20 times a month

1 a day = 30 times a month

2-3 times a day = 2.5*30 times a month = 75 times a month

4 or more times a day = 4*30 times a month = 120 times a month

Table S1. The categories of socio-economic status (SES) were based on the International Standard Classification of Occupations (ISCO) codes:

ISCO (1st digit)	*Broad Category*	*SES*	*N (%)*
0,1,2,3	0: Armed forces occupations<br>1: Managers<br>2: Professionals<br>3: Technicians and Associate Professionals	High	100 (13.6)
4,5,6	4: Clerical Support workers<br>5: Services and Sales workers<br>6: Skilled agricultural, Forestry and Fishery workers	Middle	150 (20.4)
7,8,9	7: Craft and related trades workers<br>8: Plant and Machine operators and Assemblers<br>9: Elementary occupations	Low	486 (66.0)

Table S2. Average weight estimated for each nutrient over the 100 repeated holdout WQS estimates. The set of weights are those associated to the nutrient mixture index with a protective effect on PD.

Nutrient	*Average Weight*
Fish	34.5%
Coffee	21.2%
White meat	11.8%
Dairy	8.5%
Red meat	8.1%
Carbs	7.1%
Fruit	5.5%
Vegetables	3.2%
